# Rare CNVs and Known Genes Linked to Macrocephaly: Review of Genomic Loci and Promising Candidate Genes

**DOI:** 10.3390/genes13122285

**Published:** 2022-12-04

**Authors:** Giovanna Civitate Bastos, Giovanna Cantini Tolezano, Ana Cristina Victorino Krepischi

**Affiliations:** Human Genome and Stem-Cell Research Center, Department of Genetics and Evolutionary Biology, Institute of Biosciences, University of São Paulo—106 Rua do Matão, São Paulo 05508-090, SP, Brazil

**Keywords:** CNV, macrocephaly, neurodevelopmental disorders, *TRAPPC9*, *RALGAPB*, *RBMS3*, *ZDHHC14*

## Abstract

Macrocephaly frequently occurs in single-gene disorders affecting the PI3K-AKT-MTOR pathway; however, epigenetic mutations, mosaicism, and copy number variations (CNVs) are emerging relevant causative factors, revealing a higher genetic heterogeneity than previously expected. The aim of this study was to investigate the role of rare CNVs in patients with macrocephaly and review genomic loci and known genes. We retrieved from the DECIPHER database *de novo* <500 kb CNVs reported on patients with macrocephaly; in four cases, a candidate gene for macrocephaly could be pinpointed: a known microcephaly gene–*TRAPPC9*, and three genes based on their functional roles–*RALGAPB*, *RBMS3*, and *ZDHHC14*. From the literature review, 28 pathogenic CNV genomic loci and over 300 known genes linked to macrocephaly were gathered. Among the genomic regions, 17 CNV loci (~61%) exhibited mirror phenotypes, that is, deletions and duplications having opposite effects on head size. Identifying structural variants affecting head size can be a preeminent source of information about pathways underlying brain development. In this study, we reviewed these genes and recurrent CNV loci associated with macrocephaly, as well as suggested novel potential candidate genes deserving further studies to endorse their involvement with this phenotype.

## 1. Introduction

Macrocephaly, defined as an occipitofrontal circumference (OFC) at least two standard deviations (SD) above the mean for a given age, sex, and ethnicity [1], affects about 2% of the general population and up to 5% of the children [2,3,4]. This phenotype may be driven by the expansion of the brain parenchyma, leading to a subgroup called megalencephaly, or be related to other conditions such as hydrocephalus or thickening of the frontal skull bone (cranial hyperostosis), unconnected to a primary brain development defect [5]. It can be present at birth (congenital) or be originated postnatally during the growth period (acquired), either occurring as an isolated feature (non-syndromic) or associated with other clinical signs (syndromic), including intellectual disability (ID)/neurodevelopmental delay, obesity, and overgrowth.

Because macrocephaly may indicate an underlying disorder, imaging exams, such as computerized tomography scan, head ultrasound, and magnetic resonance imaging can help to narrow the diagnosis, even in utero [6,7]. However, the etiology of most cases remains unknown, and frequently there is an absence of other significant clinical findings that could contribute to unravel the origin of this phenotype [4]. Nonetheless, it is known that several genetic syndromes have macrocephaly as a main feature, originating from *de novo* or inherited mutations, such as in Sotos and fragile X syndromes, respectively [2,3].

Most cases of macrocephaly with a known etiology are due to single-gene disorders affecting the PI3K-AKT-MTOR pathway, which directly acts on the process of brain development, including the maintenance, differentiation, and migration of neuronal progenitors, synaptogenesis, and regulation of protein translation. Other commonly affected pathways in macrocephaly cases are Ras/MAPK, that regulates cell cycle, development, senescence, transduces extracellular signs, and SHH, which takes part in the early development of the nervous system [8].

Several genes have already been linked to macrocephaly, amongst which we highlight two as examples. *PTEN* (OMIM *601728) is a tumor suppressor that interacts with several proteins within the PI3K-AKT-MTOR pathway [9], acting to restrain cell proliferation, growth, and survival [10]. *PTEN* haploinsufficiency during the early embryonic development leads to increased proliferation of neural progenitor cells and altered apoptosis that could result in macrocephaly [11,12]. Pathogenic variants in this gene are responsible for about 1/5 of the macrocephaly cases in autistic individuals, and extreme macrocephaly (>3 SD) frequently occurs due to *PTEN de novo* mutations [4,13].

The second example is the *FMR1* gene (OMIM *300805), a translational regulator associated with the fragile X syndrome. Repression of this gene in the syndrome can also result in macrocephaly due to an unbalance on ribosome biogenesis, which increases the proliferation of neural progenitor cells [14,15].

Besides single nucleotide variants, epigenetic mutations, mosaicism, and copy number variations (CNVs) are emerging as important causative factors of macrocephaly, implying a greater underlying genetic heterogeneity than previously anticipated [16,17]. For example, *AKT3* (OMIM *611223) is the main driver of the abnormalities in head size observed in the reciprocal CNVs at the 1q43-q44 region [18,19]. The AKT3 protein is essential for proper cell survival, proliferation, and growth, and it is mostly expressed in the brain. *AKT3* duplication and other gain-of-function variants increase its catalytic-kinase activity, upregulating the signaling of the PI3K-AKT pathway [19,20].

CNVs, defined as germline deletions or duplications larger than 1 kb [21], are a remarkable source of genetic variability. They account for up to 10% of the human genomic differences and can be causative of many diseases by a variety of mechanisms, affecting gene function or dosage [22]. Rare CNVs encompassing dosage-sensitive genes are frequently associated with morbid phenotypes [23], including ID and other neurodevelopmental disorders [24,25,26]. The effects of gene dosage sensitivity can be due to various factors, the most common being haploinsufficiency, when there is a minimal threshold needed for the normal functioning of the protein, affected in the disease state by loss-of-function (LoF) mutations or heterozygous deletions [23]. Sometimes, the excess of gene product in duplications can be deleterious as well, and some genes are susceptible to any modification in dosage, resulting in reciprocal CNVs displaying most commonly “mirrored” pathogenic phenotypes [27].

Although it is the most endorsed hypothesis to explain why this happens, dosage alterations are not the single mechanism through which CNVs can lead to disease. CNVs can cause a disruption within the gene, disassociate them from their regulatory sequences, or alter chromosome three-dimensional organization. Nevertheless, it is often hard to find a single dosage-sensitive gene within a CNV that encompasses several genes, because the clinically relevant CNVs are usually rare and exhibit incomplete penetrance and variable expressivity [28].

In this study, we aim to delineate the role of CNVs in macrocephaly development through evaluation of the public database DECIPHER [29], which contains information regarding CNVs and associated phenotypes, including macrocephaly. We further elaborated a comprehensive catalogue of genes and recurrent CNV syndromes known to be associated with macrocephaly.

## 2. Materials and Methods

### 2.1. DECIPHER Patients

We searched in the public data deposited in the DECIPHER database [29] for patients carrying CNVs and presenting macrocephaly among their clinical signs (up to 3 August 2021). For further analysis, we selected only cases with *de novo* CNVs with a maximum size of 500 kb, excluding all variants classified as benign and patients who carried other previously identified pathogenic variants. A CNV size of 500 kb was established as threshold to reduce the number of genes to be evaluated as candidates for the macrocephaly phenotype within each variant. Genetic and clinical data of five of the recovered DECIPHER patients were previously published [30,31,32,33,34,35]. CNVs were classified following the joint consensus recommendation of the American College of Medical Genetics and Genomics (ACMG) and the clinical genome resource (ClinGen) [36].

Genes mapped to the genomic regions of the <500 kb *de novo* CNVs were evaluated regarding their potential to contribute to the macrocephaly phenotype, using the database PubMed (https://pubmed.ncbi.nlm.nih.gov/), looking for previous association with macrocephaly or involvement in cellular processes whose imbalance could lead to an abnormal head size, such as cellular proliferation. The Human Protein Atlas (https://www.proteinatlas.org/) was also evaluated to confirm the protein expression in the brain.

### 2.2. Literature Review of Known Macrocephaly Genes and Associated CNV Syndromes

We performed an extensive review through ClinGen (https://www.clinicalgenome.org/), DECIPHER (https://www.deciphergenomics.org/), OMIM (https://www.omim.org/), and PubMed (https://pubmed.ncbi.nlm.nih.gov/) to gather updated information about the genetic mechanisms associated with macrocephaly.

Genes and CNV syndromes associated with this phenotype were filtered among the records of the OMIM database (up to 13 June 2022) according to the following criteria: (a) containing the term “macrocephaly” or “megalencephaly”, with a known molecular basis and phenotype description, or (b) containing the term “macrocephaly” or “megalencephaly”, and using the phenotype mapping key “4”, which identifies chromosomal duplication or deletion syndromes.

In the ClinGen and DECIPHER databases, we inspected all the curated recurrent CNVs recognized as dosage sensitive regions associated with clinical phenotypes [37,38], retrieving those in which macrocephaly is a typical clinical sign.

Further, we explored the PubMed repository to retrieve articles by using the terms “macrocephaly” or “megalencephaly”, aiming to complement the list of genes and CNV syndromes. This analysis was based on the evaluation of articles published in the last five years, describing genetic findings of series of patients with macrocephaly.

The collected known genes were uploaded on the WebGestalt website (http://www.webgestalt.org/) to explore the biological pathways enriched in this set (Homo sapiens; genome protein-coding genes reference list, over-representation analysis, pathway as the functional database, crossing over data with the Reactome database).

## 3. Results

### 3.1. CNVs Associated with Macrocephaly in the DECIPHER Database

**Figure 1:**
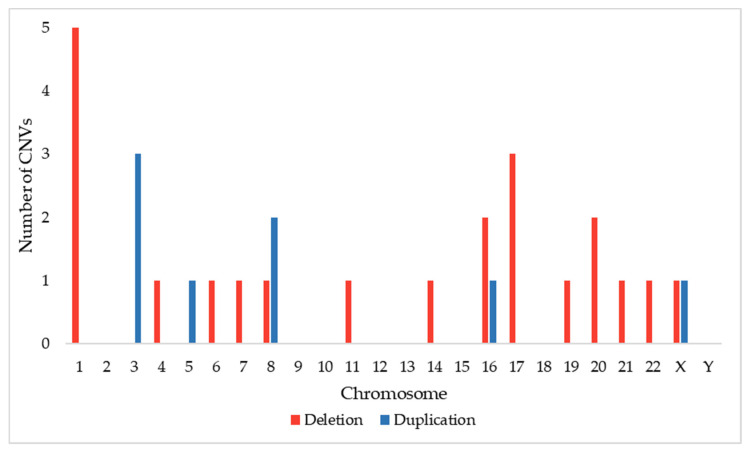
Frequency of chromosomes harboring rare *de novo* <500 kb CNVs detected in 29 macrocephalic patients described in the DECIPHER database.

We were able to retrieve information on DECIPHER about 1033 macrocephalic patients with CNV sizes ranging from 1.72 kb to 248.96 Mb (aneuploidy), with a mean size of 7.67 Mb and a median size of 1.32 Mb. For further analysis, we selected only 29 patients with *de novo* <500 kb CNVs (Figure 1).

The protein-coding genes encompassed by the CNVs were evaluated to establish a possible link with macrocephaly (Table 1). In 14 patients (~48%), the CNVs included 13 genes already known to be associated with the phenotype (*CHD8*, *GATAD2B*, *GPC3*, *KCTD13*, *KMT5B*, *KIF22*, *MAP2K2*, *MECP2*, *NF1*, *NFIA*, *NSD1*, *PUF60*, and *TCF20*), one of them (*NFIA*) observed in two cases. In total, 8 patients (~53%), amid the remaining 15, harbored a CNV that encompassed, total or partially, at least one known microcephaly gene (*ADNP*, *CACNA1G*, *DONSON*, *GRIN2A*, *ITSN1*, *MCPH1*, *RPL11*, *TBCD*, and *TRAPPC9*), for which the reported mechanism for microcephaly is mostly LoF.

We also proposed potential candidate genes in four cases (~14%) without known macrocephaly gene within the CNV (Table 2): in one case, the detected CNV encompassed a known microcephaly gene (*TRAPPC9*), and in three others, the *RALGAPB*, *RBMS3*, and *ZDHHC14* genes were highlighted, mainly based on an in silico analysis of their functional roles, as described in Table 2.

### 3.2. Literature Review of Macrocephaly Genes and Associated CNV Syndromes

We assembled a list of 341 *bona fide* genes whose association with macrocephaly has been previously corroborated, as present in a recognizable syndrome, or when at least more than one case was reported with a gene mutation linked to macrocephaly (Table S1). Aiming to unveil the main biological processes enriched for this set of genes, we performed an analysis on WebGestalt. As expected, the set of macrocephaly genes were enriched for development of the head, skull, and central nervous system and processes related to the cell cycle. In relation to neurogenesis, enriched processes included generation of new neurons, neuron projection, and gliogenesis (Figure S1).

Furthermore, we compiled 28 genomic loci with recurrent CNV syndromes that include macrocephaly among their clinical findings: 15 deletions, 11 duplications, one triplication, and one recurrent region [del/dup], as presented on Table 3. Eighteen CNV regions had an OMIM entry, four regions were exclusively described in the ClinGen or DECIPHER databases, and five regions were retrieved from the scientific literature. Seven loci were exclusively associated with macrocephaly: 1p32p31 deletion, 3q13.31 deletion, 4q32.1q32.2 triplication, 5p13 duplication, distal 7q (7q32-qter) duplication, 14q11.2 deletion, and Xq22.3 telomeric deletion. Six loci were reported to cause macrocephaly or microcephaly with the same CNV type: 2q31.2 deletion, 10p15.3 deletion, 11q deletion, 15q11q13 deletion, 17q11.2 recurrent region (del/dup), and distal 22q11.2 duplication. A particularly interesting fact is that 17 loci (65%) exhibited mirror phenotypes: they are reciprocal deletions and duplications known to originate opposite effects on head size: 1q21.1, 4pter, 5q35, 7p22.1, 7q11.23, 8p23.1, 10q22.3q23.2, 13q31.3, 15q11q13, 15q26qter, 16p11.2, 17p13.1, 17q11.2, 17q12, 17q21.31, 19p13.13, and 22q11.2.

## 4. Discussion

**Figure 2:**
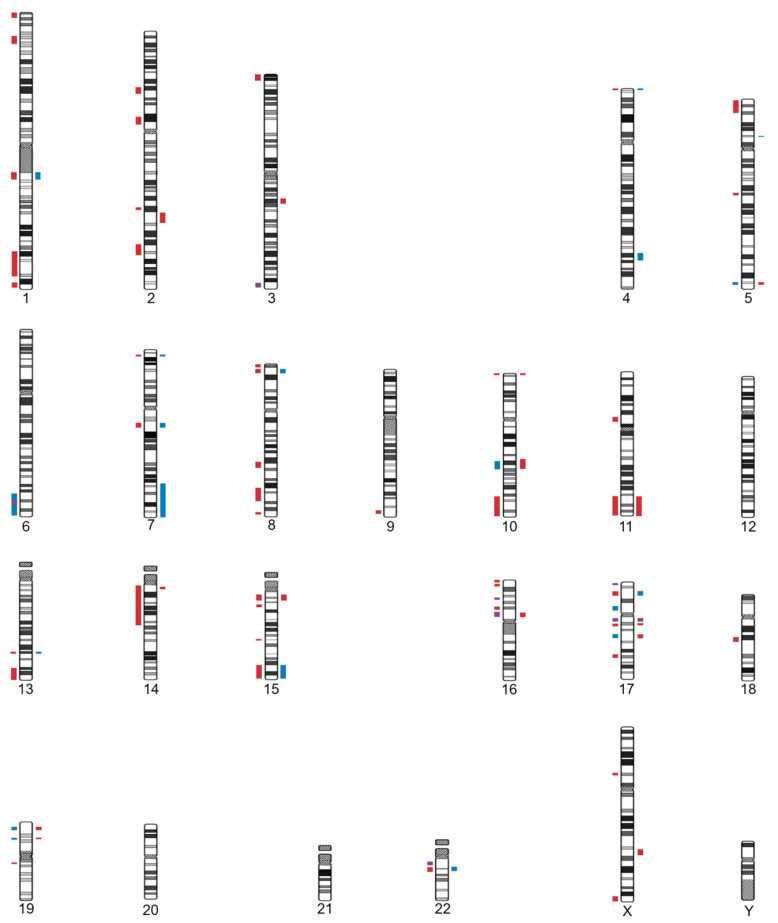
Map of the loci with CNV syndromes-deletions in red, duplications in blue, or both in purple-associated with microcephaly-*represented on the left side of the chromosomes*-or macrocephaly-*represented on the right side of the chromosomes*.

Understanding the mechanisms of brain growth and development underlying macrocephaly can shed light to the complex process of neurodevelopment [65]. CNVs affect up to 10% of the human genome and are mostly not deleterious [66,67]. Nonetheless, in neuropsychiatric disorders, such as autism and intellectual disability—which are commonly associated with alterations in head size—there is a notable enrichment of recurrent typical CNVs, resulting from nonallelic homologous recombination of hotspots flanked by paired low copy repeats [23,68]. In fact, copy number changes often can lead to protein imbalance of the affected genes, resulting in a pathogenic phenotype in case of dosage-sensitivity [23,28,66,68]. As expected, based on theory prediction and observation in model organisms, deletions (haploinsufficiency) are more common and penetrant than duplications (triplosensitivity) for extreme developmental phenotypes [28,69]; in the investigated DECIPHER cohort, over 75% of the CNVs that met our criteria were deletions. This present study and our previous review of CNVs in microcephaly [70] generated a map of CNV loci associated with alterations in head size (Figure 2). A total of 67 loci were gathered, harboring 77 CNVs (58 deletions and 19 duplications), reinforcing the relevance of CNVs, mostly deletions, in neurodevelopmental phenotypes [28,69].

The phenotypes presented by reciprocal CNVs can be allocated in four major categories: mirrored (when deletions and duplications have opposite effects), identical (both deletion and duplication result in the same phenotype spectrum), overlapping (some clinical features are present in both types of CNVs), and unique (exclusive for the deletion or duplication) [68]. Despite many cases having major driver genes responsible for the main clinical features, because of the large size of the CNVs, several genes can be affected, possibly contributing to the variability of the phenotype presented through synergistic or additive epistatic effects [66,68].

Mirror phenotypes are not universal, but frequently are present at reciprocal CNVs when head size is involved; as an example, we can cite the chromosome 5q35 region. The deletion of this region, including the *NSD1* gene, results in macrocephaly, one of the phenotypes of the Sotos syndrome, while the duplication leads to a microcephalic phenotype, likely due to gene dosage effect [71]. Identical phenotypes are probably a result of a disruption in the same developmental pathways, with either LoF mutations or overexpression and enhanced gene activity leading to similar clinical features due to downstream alterations [66,68].

Revisiting the list of genomic loci linked to macrocephaly, compiled through examination of the scientific literature available at PubMed and other aforementioned public databases, three of these categories were discernible: (a) reciprocal CNVs leading to a mirror phenotype–15 out of the 28 (~53%) known recurrent CNVs identified in this study present opposite head sizes depending on the CNV type; (b) CNVs associated exclusively with macrocephaly, constituting about 25% of the syndromes identified (seven cases), and (c) the same CNV type resulting in macro and microcephaly, as presented in the seven remained cases (25%). The latter category can be illustrated by the patient 412759, who carried a pathogenic *ADNP* intragenic deletion, leading to LoF, which has been previously associated with the ADNP syndrome, with “large head” amongst its clinical findings, as described by Li et al. (2017) [72] and Gozes (2020) [73]. On the other side, studies using animal models and mutant embryonic stem cells were able to correlate ADNP deficiency with downregulation of the homeobox gene *PAX6*, which has a crucial role in neuronal progenitor cells migration and differentiation in the developing brain and has already been described in association with microcephaly [74,75].

Through examination of the DECIPHER cohort, we observed that 14 of the 29 patients (~48%) presented a CNV encompassing a known macrocephaly gene that could explain the phenotype. Particularly, the gene *NFIA* was found to be affected by a heterozygous intragenic deletion in two patients; its haploinsufficiency is considered a main driver to the phenotypes resulting of the chromosome 1p32-p31 deletion syndrome (OMIM #613735), especially macrocephaly and intellectual disability [76].

We found a remarkably case of a conflicting phenotype in the DECIPHER patient 269967, who presented a complete deletion of the *RPL11* gene. This gene encodes a protein that is part of the large ribosomal unit, and its haploinsufficiency (amid other ribosomal proteins, such as RPL5 and RPL26, that impair the processing of pre-RNAs and the subsequent maturation of the ribosomal subunits) is the most common causative mechanism of the autosomal dominant disorder Diamond-Blackfan anemia (OMIM #612562). Almost 1/3 of the individuals with Diamond-Blackfan anemia show a degree of growth deficiency, and microcephaly is one of the present craniofacial features [77]. This is no surprise, considering that RPL11 is one of the ribosomal proteins that can interfere on the TP53-signaling pathway when in a deficiency state, leading to suppression of cell cycle progression and apoptosis due to a nucleolar stress response [78]. There is a subgroup of patients carrying chromosomal rearrangements and large deletions of other gene (RPS19) who present macrocephaly instead of microcephaly as one of the reported craniofacial abnormalities [79]; however, we found no description of *RPL11* LoF and overgrowth, as presented by the patient here discussed. Considering that *RPL11* is the only affected gene in the deleted segment, further genetic analysis would be needed to ensure if this is the only pathogenic variant carried by this patient and establish if this variant is indeed the cause of macrocephaly.

For the assessment of potential new candidate genes for macrocephaly, we evaluated the genomic content of the remaining 14 patients who did not carry CNVs encompassing known macrocephaly genes, but still presented protein-coding genes within the affected region. From this group, we were able to further cluster them in two categories based on similarities between them.

Eight DECIPHER patients presented a CNV that encompassed a known microcephaly gene; one of these patients (DECIPHER 339955) carried a partial duplication of *TRAPPC9*. Although there are duplications reported in this region in the normal population (DGV database), they do not completely overlap the distal sequence of *TRAPPC9*, which is duplicated in this patient, mainly exon 7. *TRAPPC9* LoF is associated with a rare recessive neurodevelopmental syndrome with obesity and postnatal microcephaly as the most prominent signs [80,81], the latter likely due to the role of this protein in postmitotic neurons. It acts in the vesicular protein trafficking between the Golgi apparatus and the endoplasmic reticulum, and like several genes of this group, it is related to the proper development of the nervous system. It may also play a role in the NF-κB signaling, a crucial pathway to neuronal cell differentiation and myelin formation [82,83]. Another interesting aspect is its parent-of-origin expression bias in the brain, being predominantly expressed from the maternal allele [83]. It is important to mention that the CNV data deposited in the DECIPHER is mainly based on chromosomal microarray analysis, which hampers structural evaluation of the copy number alteration. Therefore, it is not possible to determine, in case of duplications, if an intragenic or partial duplication variant is located *in tandem* or elsewhere in the genome. However, it is plausible to argue that, if the duplication is in tandem, in a direct or inverted orientation, a LoF effect would be expected, though further analysis is needed to corroborate this hypothesis.

Three patients harbored a CNV affecting non-OMIM genes (*RALGAPB*, *RBMS3* and *ZDHHC14*), whose functions, when disturbed, could potentially lead to abnormal head size. One of them carried a partial deletion of *RALGAPB*, a known tumor suppressor [84], which inhibits cell proliferation and tumor growth. Studies using animal models also emphasized the importance of the RalGAP complex for neuronal development and differentiation [85], and both knockdown and overexpression of *RALGAPB* in mammalian cells lead to an increase in mTORC1 activity [86]. This gene has little evidence for haploinsufficiency [87], but the inactivation of the multiprotein RalGAP complex has been proposed as a causal factor for microcephaly [88]. More studies are necessary to endorse *RALGAPB* as a possible candidate for macrocephaly. We observed a DECIPHER patient carrying a *RBMS3* partial duplication, similar to the *TRAPPC9* case previously mentioned. In vitro and in vivo studies demonstrated that *rbms3* inhibits cell proliferation and promotes apoptosis due to regulation of gene transcription or RNA metabolism, and its expression is reduced in several cancers [89,90]. Defects in RNA-binding proteins, such as the aforementioned, may lead to craniofacial abnormalities. Jayasena & Bronner (2012) [39] performed a study to analyze the consequences of *rbms3* LoF during zebrafish development; the mutants had a variety of abnormalities when compared to the wild type, including smaller body size and craniofacial defects due to improper cartilage formation. The reported association of rbms3 with craniofacial abnormalities in animal models and *RBMS3* pHaplo of 0.89 (an ensemble machine-learning model, designed by Collins (2022) [27], that reflects the probability of haploinsufficiency for autosomal genes) indicate that this gene could be a strong candidate for the macrocephaly phenotype, even though further analysis is required to validate this assumption. *ZDHHC14* expression is also reduced in several cancer types, including brain tumors; induced in vitro overexpression in gastric cancer cell lines promoted cancer cell migration and cell attachment, in addition to stimulating cell invasion [91]. In vitro studies by Yeste-Velasco et al. (2014) [92] demonstrated that whereas the overexpression promoted apoptosis through activating of the classic caspase-dependent pathway, heterozygous deletion increased colony formation. Therefore, *RBMS3* and *ZDHHC14* are both classified as tumor suppressor genes, and the CNVs identified in these DECIPHER patients have a probable LoF effect (intragenic duplication and entire gene deletion, respectively). Considering that the reduced expression of these two genes is reported to increase cell proliferation and/or inhibit apoptosis, they are interesting candidates and additional studies are required to provide functional support to their potential causal correlation with macrocephaly.

Finally, we found two CNVs encompassing non-coding genes that are worth mentioning. One of them was a deletion including only part of the sequence of the long intergenic non-protein coding RNA 1162 (LINC01162), mapped to 7p15.3 (patient 288535); however, despite being a validated lncRNA, there is no information regarding its function. The second CNV was an 8p23.1 duplication (patient 314265), which encompassed three protein coding genes, including part of the known microcephaly gene *MCPH1*, and the full sequence of its antisense lncRNA (MCPH1-AS1), a validated gene with high expression in brain tissues [93]. It is difficult to anticipate the potential impact of CNVs harboring lncRNAs; both variants were classified as likely benign according to the clinical guidelines.

Studies focusing on pathogenic variants disrupting the mechanisms that control head size are an extremely important source of information about biological pathways underlying these processes. This study reviewed the genes and CNV loci previously associated with macrocephaly in the literature as well as suggested novel potential candidate genes deserving further evaluation.

## Figures and Tables

**Table 1:** Description of the CNV data from the 29 macrocephalic patients with *de novo* <500 kb CNVs reported in the DECIPHER database and encompassed genes (in **bold,** known macrocephaly genes; ↓ known microcephaly genes; in red, potential candidate genes for macrocephaly).

DECIPHER ID	Chromosomal Microarray Analysis (CMA)					Genotype-Phenotype Correlation	References
	Cytoband	Genomic Coordinates (GRCh38)	CNV Type	Classification	Size (kb)	Included Genes	
						(Protein Coding)	
281752	1q21.3	1:153818614-153823418	Deletion ^P^	Pathogenic	5	***GATAD2B***	
269967	1p36.11	1:23691668-23696616	Deletion ^P^	Pathogenic	5	*RPL11* ↓	
358646	1p31.3	1:61091785-61304381	Deletion ^P^	Pathogenic	213	***NFIA***	
288170	1p31.3	1:61151026-61379931	Deletion ^VUS^	Pathogenic	229	***NFIA***	
360889	1p36.33	1:917483-1282652	Deletion ^P^	VUS	365	*AGRN*/*B3GALT6*/*C1QTNF12*/*C1orf159*/*HES4*/*ISG15*/*KLHL17*/*NOC2L*/*PERM1*/*PLEKHN1*/*RNF223*/*SAMD11*/*SCNN1D*/*SDF4*/*TNFRSF18*/*TNFRSF4*/*TTLL10*/*UBE2J2*	
249115	3q25.33	3:159979210-160186591	Duplication ^U^	Likely benign	207	*IL12A*	[32]
	Xq28	X:154032185-154347882	Duplication ^U^	Pathogenic	316	***MECP2*** ↓/*OPN1LW*/*OPN1MW*/*OPN1MW2*/*OPN1MW3*/*TEX28*/*TKTL1*	
1376	3q27.2	3:185411752-185567638	Duplication ^U^	VUS	156	*LIPH*/*MAP3K13*/*TMEM41A*	
370137	3p24.1	3:29248723-29450441	Duplication ^LP^	VUS	202	*RBMS3*	
289221	4q22.2	4:93137138-93292777	Deletion ^VUS^	VUS	156	*GRID2*	
267309	5q35.3	5:177176839-177225635	Duplication ^U^	VUS	49	***NSD1*** ↓	
376373	6q25.3	6:157361157-157681369	Deletion ^VUS^	VUS	320	*ZDHHC14*	
288535	7p15.3	7:20953880-21052118	Deletion ^VUS^	Likely benign	98	*-*	
339955	8q24.3	8:140361494-140527307	Duplication ^VUS^	VUS	166	*AGO2*/*CHRAC1*/*TRAPPC9* ↓	
371384	8q24.3	8:143809435-143822571	Deletion ^P^	Pathogenic	13	***PUF60*** ↓/*SCRIB ↓*	[30]
314265	8p23.1	8:6518891-6801141	Duplication ^P^	VUS	282	*MCPH1* ↓/*ANGPT2*/*AGPAT5*	
251808	11q13.2	11:68120554-68519565	Deletion ^U^	Pathogenic	399	*LRP5*/***KMT5B***/*CHKA*/*C11ORF24*/*PPP6R3*	
976	14q11.2	14:21309552-21460179	Deletion ^U^	Pathogenic	151	***CHD8***/*RAB2B*/*RPGRIP1*/*SUPT16H*	[35]
253656	16p11.2	16:29837876-30179218	Deletion ^U^	Pathogenic	341	*TAOK2*/*TLCD3B*/*PPP4C*/*CDIPT*/*SEZ6L2*/*YPEL3*/*MAPK3*/*C16orf92*/*INO80E*/*TBX6*/*DOC2A/ALDOA/ASPHD1*/***KCTD13*** ↓/*TMEM219*/*HIRIP3*/*MVP*/*GDPD3*	
285342	16p13.2	16:9732634-9770161	Deletion ^LP^	Pathogenic	38	*GRIN2A* ↓	
301907	17q11.2	17:31334752-31340757	Deletion ^P^	Pathogenic	6	***NF1*** ↓	
287501	17q21.33	17:50600602-50604425	Deletion ^U^	Likely benign	4	*CACNA1G* ↓	[31,33]
368685	17q25.3	17:82821407-83086677	Deletion ^VUS^	Likely benign	265	*ZNF750*/*METRNL*/*TBCD* ↓/*B3GNTL1*	
300874	19p13.3	19:3979570-4131262	Deletion ^P^	Pathogenic	152	*EEF2*/*PIAS4*/***MAP2K2***/*ZBTB7A*	
270687	20q11.23	20:38565558-38765539	Deletion ^U^	VUS	200	*RALGAPB*/*SLC32A1*/*ADIG*/*ARHGAP40*/*ACTR5*	[34]
412759	20q13.13	20:50891372-50893349	Deletion ^P^	Pathogenic	2	*ADNP* ↓	
249393	21q22.11	21:33581937-33883538	Deletion ^U^	VUS	302	*ITSN1*↓*/DONSON* ↓/*CRYZL1*	
259449	22q13.2	22:41743074-42171084	Deletion ^VUS^	Pathogenic	428	*CCDC134*/*CENPM*/*CYP2D6*/*CYP2D7*/*MEI1*/*NAGA*/*NDUFA6*/*PHETA2*/*SEPTIN3*/*SHISA8*/*SMDT1*/*SREBF2*/***TCF20***/*TNFRSF13C*/*WBP2NL*	
270868	Xq26.2	X:133552850-134042983	Deletion ^U^	Pathogenic	490	***GPC3***	
	16p11.2	16:29552664-30095687	Duplication ^P^		543	*ALDOA*/*ASPHD1*/*C16orf54*/*C16orf92*/*CDIPT*/*DOC2A*/*HIRIP3*/*INO80E*/***KCTD13*** ↓/***KIF22***/*MAS*/*MVP*/*PAGR1*/*PPP4C*/*PRRT2*/*QPRT*/*SEZ6L2*/*SPN*/*TAOK2*/*TBX6*/*TLCD3B*/*TMEM219*/*YPEL3*/*ZG16*	

^P^: classified as pathogenic on DECIPHER; ^LP^: classified as likely pathogenic on DECIPHER; ^VUS^: classified as variant of uncertain significance on DECIPHER; ^U^: unclassified on DECIPHER; VUS: variant of unknown significance.

**Table 2:** Potential candidate genes for macrocephaly and their biological functions.

Gene	Function *	Brain Expression ꜝ	Type of Variant (Probable Effect)	Reference
*TRAPPC9* ↓	May function in neuronal cells differentiation. LoF associated with microcephaly ^r^	Yes	Partial duplication (unknown)	(OMIM #613192)
*RALGAPB*	*RALGAPB* plays an essential role in mitosis by controlling the spatial and temporal activation of RAL GTPases in the spindle assembly checkpoint (SAC) and cytokinesis	Yes	Partial deletion (unknown)	(OMIM *618833)
*RBMS3*	Rbms3 was shown to exhibit tumor suppressor function via regulation of *c*-*Myc* and to bind/stabilize RNA in vitro. In zebrafish, LoF disrupts craniofacial development; not previously related to human diseases	Yes	Intragenic duplication (LoF)	(OMIM *300027) [39]
*ZDHHC14*	Overexpression of *ZDHHC14* reduces cell viability and induces apoptosis by activating a classic caspase-dependent pathway, whereas heterozygous knockout of *ZDHHC14* increased colony formation ability of cells.	Yes	Entire deletion (LoF)	(OMIM *619295)

↓: known microcephaly genes; * Described in UniProt; ꜝ According to the Human Protein Atlas.

**Table 3:** Twenty-eight recognized loci harboring deletion/duplication syndromes associated with macrocephaly (OMIM number, genomic coordinates, inheritance pattern, and ClinGen/DECIPHER data are shown, when applicable).

Condition	OMIM #	Genomic Coordinates (hg38)	Inheritance Pattern	Association with Head Size	ClinGen/ DECIPHER	Additional References
Chromosome 1p32-p31 deletion syndrome	613735	chr1:58193565-63125273	Incomplete penetrance	Macrocephaly	…	[40]
Chromosome 1q21.1 duplication syndrome	612475	chr1:147105904-147917509	Incomplete penetrance	Macrocephaly in duplication/Microcephaly in deletion (#612474)	Yes	[41]
Chromosome 2q31.2 deletion syndrome	612345	chr2:177100000-179700000	Incomplete penetrance	Macrocephaly/Microcephaly	...	-
Chromosome 3q13.31 deletion syndrome	615433	chr3:113700000-117600000	de novo	Macrocephaly in deletion/Normal OFC in duplication	...	[42,43]
Chromosome 4pter duplication syndrome	N/A	chr4:337779-2009235	de novo	Macrocephaly in duplication/Microcephaly in deletion	Yes	[44]
Chromosome 4q32.1-q32.2 triplication syndrome	613603	chr4:154600000-163600000	de novo	Macrocephaly	...	[45]
Chromosome 5p13 duplication syndrome	613174	chr5:36845462–37231819	de novo	Macrocephaly	...	[46]
Chromosome 5q35 deletion syndrome (Sotos 1)	117550	chr5:176297633-177625115	de novo	Macrocephaly in deletion/Microcephaly in duplication	Yes	[47]
Chromosome 7p22.1 duplication syndrome	N/A	chr7:5527147-5530600	de novo	Relative macrocephaly in duplication/Microcephaly in deletion	...	[48]
Chromosome 7q11.23 duplication syndrome	609757	chr7: 73330452-74728172	Mostly de novo	Macrocephaly in duplication/Microcephaly in deletion (#194050)	Yes	-
Chromosome distal 7q (7(q32→qter)) duplication syndrome	N/A	chr7:128308047– 159119707	Incomplete penetrance (can be inherited)	Macrocephaly	Yes ^!^	[49]
Chromosome 8p23.1 duplication syndrome	N/A	chr8:8242542-11908820	de novo	Macrocephaly in duplication/Microcephaly in deletion	Yes	[28,50,51]
Chromosome 10p15.3 deletion syndrome	N/A	chr10:171237-2880776	Incomplete penetrance (can be inherited)	Macrocephaly/Microcephaly	...	[52,53,54]
Chromosome 10q22.3-q23.2 deletion syndrome	612242	chr10:80300000-95300000	de novo	Macrocephaly in deletion/Microcephaly in duplication	Yes	-
Chromosome 11q deletion syndrome (Jacobsen)	147791	chr11:114600000-135086622	de novo	Macrocephaly/Microcephaly	Yes	-
Chromosome 13q31.3 microduplication syndrome	N/A	chr13:91337007-91852603	de novo	Macrocephaly in duplication/Microcephaly in deletion	...	[55]
Chromosome 14q11.2 microdeletion syndrome	N/A	chr14:21359783-21393052	de novo	Macrocephaly	…	[56]
Chromosome 15q11q13 deletion (PWS)	176270	chr15:22832519-28379874	de novo	Macro-microcephaly in PWS/Microcephaly in AS	Yes	-
Chromosome 15q26qter duplication syndrome	612626	chr15:88500000-101991189	de novo	Macrocephaly in duplication/Microcephaly in deletion	Yes	[57]
Chromosome 16p11.2 deletion syndrome, 593kb	611913	chr16:29595531-30188534	Incomplete penetrance (can be inherited)	Macrocephaly in deletion/Microcephaly in duplication	Yes	[58]
Chromosome 17p13.1 duplication syndrome	N/A	chr17:7584958-8092957	de novo	Macrocephaly in duplication/Microcephaly in deletion	...	[45,59]
Chromosome 17q11.2 recurrent region 1.4Mb (del/dup)	613675 618874	chr17:30780079-31937008	Del-de novo; Dup-incomplete penetrance (can be inherited)	Macrocephaly/Microcephaly	Yes	-
Chromosome 17q12 deletion syndrome	614527	chr17:36458167-37854616	Mostly de novo	Macrocephaly in deletion/Microcephaly in duplication	Yes	[60]
Chromosome 17q21.31 deletion syndrome	610443	chr17:45627800-46087514	Description of inherited cases	Macrocephaly in deletion/Microcephaly in duplication (#613533)	Yes	[61]
Chromosome 19p13.13 deletion syndrome	613638	chr19:12821186-13132186	de novo	Macrocephaly in deletion (#613638)/Microcephaly in duplication	...	[45]
Chromosome 19p13.3 microdeletion syndrome	N/A	chr19:2329321-4996917	Description of inherited cases, although the majority are de novo	Macrocephaly in deletion/Microcephaly in duplication	…	[62,63]
Chromosome 22q11.2 duplication syndrome, distal	N/A	chr22:21562828-23306924	Incomplete penetrance (can be inherited)	Macro-microcephaly in duplication/Microcephaly in deletion	Yes	[64]
Chromosome Xq22.3 telomeric deletion syndrome (AMME)	300194	chrX:104500000-109400000	Dominant X-linked (description of inherited cases)	Macrocephaly	...	-

N/A: Not Available; ^!^ includes https://dosage.clinicalgenome.org/region_search_38.cgi?loc=chr7:128,667,993-159,327,017.

## Data Availability

This study makes use of data generated by the DECIPHER community. A full list of centers who contributed to the generation of the data is available from https://deciphergenomics.org/about/stats and via email from contact@deciphergenomics.org. Funding for the DECIPHER project was provided by Wellcome. Those who carried out the original analysis and collection of the data bear no responsibility for the further analysis or interpretation of them.

## Supplementary Materials

The following supporting information can be downloaded at: https://www.mdpi.com/article/10.3390/genes13122285/s1, Table S1: Genes with a recognizable association with macrocephaly, retrieved from OMIM and the scientific literature; Figure S1: Enriched biological processes of the gene set related to macrocephaly.
