# Supplementary material for: Rare CNVs and Known Genes Linked to Macrocephaly: Review of Genomic Loci and Promising Candidate Genes
Source: Genes (Basel). 2022 Dec 4;13(12):2285. doi: 10.3390/genes13122285 (PMC9778424; doi:10.3390/genes13122285)

**Table S1.** Genes with a recognizable association with macrocephaly, retrieved from OMIM and the scientific literature.

| Gene | Gene/Locus name | Cytogenetic location (hg38) |
|---|---|---|
| *ABCC9* | ATP binding cassette subfamily C member 9 | **12p12.1** |
| *ACAN* | Aggrecan | **15q26.1** |
| *ACER3* | Alkaline ceramidase 3 | **11q13.5** |
| *ADK* | Adenosine kinase | **10q22.2** |
| *ADNP* | Activity dependent neuroprotector homeobox | **20q13.13** |
| *AKT1* | AKT serine/threonine kinase 1 | **14q32.33** |
| *AKT3* | AKT serine/threonine kinase 3 | **1q43-q44** |
| *ALKBH8* | AlkB homolog 8, tRNA methyltransferase | **11q22.3** |
| *AMER1* | APC membrane recruitment protein 1 | **Xq11.2** |
| *AMPD1* | Adenosine monophosphate deaminase 1 | **1p13.2** |
| *ANKH* | ANKH inorganic pyrophosphate transport regulator | **5p15.2** |
| *ANKRD17* | Ankyrin repeat domain 17 | **4q13.3** |
| *ANTXR1* | Anthrax toxin receptor 1 | **2p13.3** |
| *APC2* | APC regulator of WNT signaling pathway 2 | **19p13.3** |
| *APOC2* | Apolipoprotein C-II | **19q13.32** |
| *ARSB* | Arylsulfatase B | **5q14.1** |
| *ARSK* | Arylsulfatase family member K | **5q15** |
| *ASH1L* | ASH1 like histone lysine methyltransferase | **1q22** |
| *ASPA* | aspartoacylase | **17p13.2** |
| *ASXL2* | ASXL transcriptional regulator 2 | **2p23.3** |
| *AUTS2* | Activator of transcription and developmental regulator AUTS2 | **7q11.22** |
| *B3GLCT* | Beta 3-glucosyltransferase | **13q12.3** |
| *B4GALT1* | Beta-1,4-galactosyltransferase 1 | **9p21.1** |
| *B4GALT7* | Beta-1,4-galactosyltransferase 7 | **5q35.3** |
| *BAP1* | BRCA1 associated protein 1 | **3p21.1** |
| *BGN* | Biglycan | **Xq28** |
| *BICD2* | BICD cargo adaptor 2 | **9q22.31** |
| *BICRA* | BRD4 interacting chromatin remodeling complex associated protein | **19q13.33** |
| *BLTP1* | Bridge-like lipid transfer protein family member 1 | **4q27** |
| *BRAF* | B-Raf proto-oncogene, serine/threonine kinase | **7q34** |
| *BRWD3* | Bromodomain and WD repeat domain containing 3 | **Xq21.1** |
| *C12orf57* | Chromosome 12 open reading frame 57 | **12p13.31** |
| *CACNA1E* | Calcium voltage-gated channel subunit alpha1 E | **1q25.3** |
| *CAMK2G* | Calcium/calmodulin dependent protein kinase II gamma | **10q22.2** |
| *CANT1* | Calcium activated nucleotidase 1 | **17q25.3** |
| *CASK* | Calcium/calmodulin dependent serine protein kinase | **Xp11.4** |
| *CBL* | Cbl proto-oncogene | **11q23.3** |
| *CCND2* | Cyclin D2 | **12p13.32** |
| *CCNK* | Cyclin K | **14q32.2** |
| *CDC42BPB* | CDC42 binding protein kinase beta | **14q32.32** |
| *CDH2* | Cadherin 2 | **18q12.1** |
| *CDKN1C* | Cyclin-dependent kinase inhibitor 1C | **11p15.4** |
| *CEP120* | Centrosomal protein, 120kD | **5q23.2** |
| *CHD1* | Chromodomain helicase DNA binding protein-1 | **5q15-q21.1** |
| *CHD3* | Chromodomain helicase DNA binding protein-3 | **17p13.1** |
| *CHD4* | Chromodomain helicase DNA-binding protein-4 | **12p13.31** |
| *CHD8* | Chromodomain helicase DNA-binding protein 8 | **14q11.2** |
| *COL2A1* | Collagen II, alpha-1 polypeptide | **12q13.11** |
| *COL3A1* | Collagen III, alpha-1 polypeptide | **2q32.2** |
| *CPE* | Carboxypeptidase E | **4q32.3** |
| *CRPPA* | CDP-L-ribitol pyrophosphorylase A | **7p21.2** |
| *CSF1R* | Colony stimulating factor 1 receptor | **5q32** |

| | | |
|---|---|---|
| *SGALNACT1* | Chondroitin sulfate N-acetylgalactosaminyltransferase 1 | **8p21.3** |
| *CSNK2A1* | Casein kinase 2 alpha 1 | **20p13** |
| *CUL4B* | Cullin 4B | **Xq24** |
| *CUL7* | Cullin 7 | **6p21.1** |
| *CWC27* | CWC27 spliceosome associated cyclophilin | **5q12.3** |
| *D2HGDH* | D-2-hydroxyglutarate dehydrogenase | **2q37.3** |
| *DAG1* | Dystroglycan 1 | **3p21.31** |
| *DDX3X* | DEAD-box helicase 3 X-linked | **Xp11.4** |
| *DEAF1* | DEAF1 transcription factor | **11p15.5** |
| *DEPDC5* | DEP domain containing 5 | **22q12.2-q12.3** |
| *DHCR24* | 24-dehydrocholesterol reductase | **1p32.3** |
| *DICER1* | Dicer 1, ribonuclease III | **14q32.13** |
| *DIS3L2* | DIS3 like 3'-5' exoribonuclease 2 | **2q37.1** |
| *DLX3* | Distal-less homeobox 3 | **17q21.33** |
| *DMXL2* | Dmx like 2 | **15q21.2** |
| *DNAJC21* | DnaJ heat shock protein family (Hsp40) member C21 | **5p13.2** |
| *DNM1* | Dynamin 1 | **9q34.11** |
| *DNMT3A* | DNA methyltransferase 3 alpha | **2p23.3** |
| *DOCK3* | Dedicator of cytokinesis 3 | **3p21.2** |
| *DOCK6* | Dedicator of cytokinesis 6 | **19p13.2** |
| *DPYSL5* | Dihydropyrimidinase-like 5 | **2p23.3** |
| *DVL1* | Dishevelled segment polarity protein 1 | **1p36.33** |
| *DVL3* | Dishevelled segment polarity protein 3 | **3q27.1** |
| *DYNC2I1* | Dynein 2 intermediate chain 1 | **7q36.3** |
| *DYNC2LI1* | Dynein cytoplasmic 2 light intermediate chain 1 | **2p21** |
| *EBP* | EBP cholestenol delta-isomerase | **Xp11.23** |
| *EED* | Embryonic ectoderm development | **11q14.2** |
| *EHMT1* | Euchromatic histone lysine methyltransferase 1 | **9q34.3** |
| *EIF2B1* | Eukaryotic translation initiation factor 2B subunit alpha | **12q24.31** |
| *EIF2B2* | Eukaryotic translation initiation factor 2B subunit beta | **14q24.3** |
| *EIF2B3* | Eukaryotic translation initiation factor 2B subunit gamma | **1p34.1** |
| *EIF2B4* | Eukaryotic translation initiation factor 2B subunit delta | **2p23.3** |
| *EIF2B5* | Eukaryotic translation initiation factor 2B subunit epsilon | **3q27.1** |
| *EML1* | EMAP like 1 | **14q32.2** |
| *ERF* | ETS2 repressor factor | **19q13.2** |
| *ETFA* | Electron transfer flavoprotein subunit alpha | **15q24.2-q24.3** |
| *ETFB* | Electron transfer flavoprotein subunit beta | **19q13.41** |
| *ETFDH* | Electron transfer flavoprotein dehydrogenase | **4q32.1** |
| *EXT2* | Exostosin glycosyltransferase 2 | **11p11.2** |
| *EZH2* | Enhancer of zeste 2 polycomb repressive complex 2 subunit | **7q36.1** |
| *FAM111A* | FAM111 trypsin like peptidase A | **11q12.1** |
| *FAM149B1* | Family with sequence similarity 149 member B1 | **10q22.2** |
| *FAM20C* | FAM20C golgi associated secretory pathway kinase | **7p22.3** |
| *FAR1* | Fatty acyl-CoA reductase 1 | **11p15.3** |
| *FBN1* | Fibrillin 1 | **15q21.1** |
| *FBXW11* | F-box and WD repeat domain containing 11 | **5q35.1** |
| *FGFR1* | Fibroblast growth factor receptor 1 | **8p11.23** |
| *FGFR2* | Fibroblast growth factor receptor 2 | **10q26.13** |
| *FGFR3* | Fibroblast growth factor receptor 3 | **4p16.3** |
| *FH* | Fumarate hydratase | **1q43** |
| *FIBP* | FGF1 intracellular binding protein | **11q13.1** |
| *FKTN* | Fukutin | **9q31.2** |
| *FLNA* | Filamin A | **Xq28** |
| *FMR1* | Fragile X messenger ribonucleoprotein 1 | **Xq27.3** |
| *FOXP1* | Forkhead box P1 | **3p13** |
| *GABBR2* | Gamma-aminobutyric acid type B receptor subunit 2 | **9q22.33** |
| *GATAD2B* | GATA zinc finger domain containing 2B | **1q21.3** |
| *GCDH* | Glutaryl-coa dehydrogenase | **19p13.13** |

| | | |
|---|---|---|
| *GDF6* | Growth differentiation factor 6 | **8q22.1** |
| *GFAP* | Glial fibrillary acidic protein | **17q21.31** |
| *GJA1* | Gap junction protein alpha 1 | **6q22.31** |
| *GLI2* | GLI family zinc finger 2 | **2q14.2** |
| *GLI3* | GLI family zinc finger 3 | **7p14.1** |
| *GNAI1* | G protein subunit alpha i1 | **7q21.11** |
| *GNAI3* | G protein subunit alpha i3 | **1p13.3** |
| *GNAQ* | G protein subunit alpha q | **9q21.2** |
| *GNE* | Glucosamine (UDP-N-acetyl)-2-epimerase/N-acetylmannosamine kinase | **9p13.3** |
| *GPC3* | Glypican 3 | **Xq26.2** |
| *GPC4* | Glypican 4 | **Xq26.2** |
| *GPSM2* | G protein signaling modulator 2 | **1p13.3** |
| *GRIA3* | Glutamate ionotropic receptor AMPA type subunit 3 | **Xq25** |
| *GRIN2B* | Glutamate ionotropic receptor NMDA type subunit 2B | **12p13.1** |
| *GUSB* | Glucuronidase beta | **7q11.21** |
| *H1-4* | H1.4 linker histone, cluster member | **6p22.2** |
| *H3-3A* | H3.3 histone A | **1q42.12** |
| *H3-3B* | H3.3 histone B | **17q25.1** |
| *HADHA* | oxyacyl-coa dehydrogenase trifunctional multienzyme complex subunit alpha | **2p23.3** |
| *HDAC6* | Histone deacetylase 6 | **Xp11.23** |
| *HECW2* | HECT, C2 and WW domain containing E3 ubiquitin protein ligase 2 | **2q32.3** |
| *HEPACAM* | Hepatic and glial cell adhesion molecule | **11q24.2** |
| *HERC1* | CT and RLD domain containing E3 ubiquitin protein ligase family member 1 | **15q22.31** |
| *HEXB* | Hexosaminidase subunit beta | **5q13.3** |
| *HMGA2* | High mobility group AT-hook 2 | **12q14.3** |
| *HRAS* | HRas proto-oncogene, GTPase | **11p15.5** |
| *HSD17B4* | Hydroxysteroid 17-beta dehydrogenase 4 | **5q23.1** |
| *HUWE1* | HECT, UBA and WWE domain containing E3 ubiquitin protein ligase 1 | **Xp11.22** |
| *HYLS1* | HYLS1 centriolar and ciliogenesis associated | **11q24.2** |
| *IDS* | Iduronate 2-sulfatase | **Xq28** |
| *IDUA* | Alpha-L-iduronidase | **4p16.3** |
| *IFITM5* | Interferon induced transmembrane protein 5 | **11p15.5** |
| *IFT43* | Intraflagellar transport 43 | **14q24.3** |
| *IFT74* | Intraflagellar transport 74 | **9p21.2** |
| *IFT81* | Intraflagellar transport 81 | **12q24.11** |
| *IGBP1* | Immunoglobulin binding protein 1 | **Xq13.1** |
| *IGF2* | Insulin like growth factor 2 | **11p15.5** |
| *IL6ST* | Interleukin 6 cytokine family signal transducer | **5q11.2** |
| *INPP5E* | Inositol polyphosphate-5-phosphatase E | **9q34.3** |
| *INPPL1* | Inositol polyphosphate phosphatase like 1 | **11q13.4** |
| *ITCH* | Itchy E3 ubiquitin protein ligase | **20q11.22** |
| *KCNH1* | Potassium voltage-gated channel subfamily H member 1 | **1q32.2** |
| *KCNJ8* | Potassium inwardly rectifying channel subfamily J member 8 | **12p12.1** |
| *KCTD13* | Potassium channel tetramerization domain containing 13 | **16p11.2** |
| *KDM1A* | Lysine demethylase 1A | **1p36.12** |
| *KDM5B* | Lysine demethylase 5B | **1q32.1** |
| *KDM5C* | Lysine demethylase 5C | **Xp11.22** |
| *KIAA0753* | KIAA0753 | **17p13.1** |
| *KIDINS220* | Kinase D interacting substrate 220 | **2p25.1** |
| *KIF22* | Kinesin family member 22 | **16p11.2** |
| *KIF7* | Kinesin family member 7 | **15q26.1** |
| *KMT2E* | Lysine methyltransferase 2E (inactive) | **7q22.3** |
| *KMT5B* | Lysine methyltransferase 5B | **11q13.2** |
| *KPTN* | Kaptin, actin binding protein | **19q13.32** |
| *KRAS* | KRAS proto-oncogene, GTPase | **12p12.1** |
| *L1CAM* | L1 cell adhesion molecule | **Xq28** |
| *L2HGDH* | L-2-hydroxyglutarate dehydrogenase | **14q21.3** |
| *LAMB1* | Laminin subunit beta 1 | **7q31.1** |

| | | |
|---|---|---|
| ***LBR*** | Lamin B receptor | **1q42.12** |
| ***LRP2*** | LDL receptor related protein 2 | **2q31.1** |
| ***MAB21L2*** | mab-21 like 2 | **4q31.3** |
| ***MADD*** | MAP kinase activating death domain | **11p11.2** |
| ***MAN1B1*** | Mannosidase alpha class 1B member 1 | **9q34.3** |
| ***MAN2B1*** | Mannosidase alpha class 2B member 1 | **19p13.13** |
| ***MAN2C1*** | Mannosidase alpha class 2C member 1 | **15q24.2** |
| ***MAP2K1*** | Mitogen-activated protein kinase kinase 1 | **15q22.31** |
| ***MAP2K2*** | Mitogen-activated protein kinase kinase 2 | **19p13.3** |
| ***MCCC2*** | Methylcrotonyl-coa carboxylase subunit 2 | **5q13.2** |
| ***MCEE*** | Methylmalonyl-coa epimerase | **2p13.3** |
| ***MECP2*** | Methyl-cpg binding protein 2 | **Xq28** |
| ***MED12*** | Mediator complex subunit 12 | **Xq13.1** |
| ***MESD*** | Mesoderm development LRP chaperone | **15q25.1** |
| ***MGAT2*** | lpha-1,6-mannosyl-glycoprotein 2-beta-N-acetylglucosaminyltransferase | **14q21.3** |
| ***MITF*** | Melanocyte inducing transcription factor | **3p13** |
| ***MLC1*** | Modulator of VRAC current 1 | **22q13.33** |
| ***MOCS1*** | Molybdenum cofactor synthesis 1 | **6p21.2** |
| ***MOCS2*** | Molybdenum cofactor synthesis 2 | **5q11.2** |
| ***MPDZ*** | Multiple PDZ domain crumbs cell polarity complex component | **9p23** |
| ***MRAS*** | Muscle RAS oncogene homolog | **3q22.3** |
| ***MSL3*** | MSL complex subunit 3 | **Xp22.2** |
| ***MTOR*** | Mechanistic target of rapamycin kinase | **1p36.22** |
| ***MTM1*** | Myotubularin 1 | **Xq28** |
| ***MUSK*** | Muscle associated receptor tyrosine kinase | **9q31.3** |
| ***MYCN*** | MYCN proto-oncogene, bHLH transcription factor | **2p24.3** |
| ***MYH8*** | Myosin heavy chain 8 | **17p13.1** |
| ***MYMK*** | Myomaker, myoblast fusion factor | **9q34.2** |
| ***NBPF1*** | NBPF member 1 | **1p36.13** |
| ***NDN*** | Necdin, MAGE family member | **15q11.2** |
| ***NDUFAF3*** | NADH:ubiquinone oxidoreductase complex assembly factor 3 | **3p21.31** |
| ***NDUFAF5*** | NADH:ubiquinone oxidoreductase complex assembly factor 5 | **20p12.1** |
| ***NDUFS4*** | NADH:ubiquinone oxidoreductase subunit S4 | **5q11.2** |
| ***NDUFV1*** | NADH:ubiquinone oxidoreductase core subunit V1 | **11q13.2** |
| ***NEK1*** | NIMA related kinase 1 | **4q33** |
| ***NF1*** | Neurofibromin 1 | **17q11.2** |
| ***NFIA*** | Nuclear factor I A | **1p31.3** |
| ***NFIB*** | Nuclear factor I B | **9p23-p22.3** |
| ***NFIX*** | Nuclear factor I X | **19p13.13** |
| ***NKX3-2*** | NK3 homeobox 2 | **4p15.33** |
| ***NONO*** | Non-POU domain containing octamer binding | **Xq13.1** |
| ***NR2F1*** | Nuclear receptor subfamily 2 group F member 1 | **5q15** |
| ***NRAS*** | NRAS proto-oncogene, GTPase | **1p13.2** |
| ***NSD1*** | Nuclear receptor binding SET domain protein 1 | **5q35.3** |
| ***NXN*** | Nucleoredoxin | **17p13.3** |
| ***OBSL1*** | Obscurin like cytoskeletal adaptor 1 | **2q35** |
| ***ODC1*** | Ornithine decarboxylase 1 | **2p25.1** |
| ***OFD1*** | OFD1 centriole and centriolar satellite protein | **Xp22.2** |
| ***OPHN1*** | Oligophrenin 1 | **Xq12** |
| ***OTUD5*** | OTU deubiquitinase 5 | **Xp11.23** |
| ***P4HB*** | Prolyl 4-hydroxylase subunit beta | **17q25.3** |
| ***PAK1*** | P21 (RAC1) activated kinase 1 | **11q13.5-q14.1** |
| ***PAK3*** | P21 (RAC1) activated kinase 3 | **Xq23** |
| ***PAM16*** | Presequence translocase associated motor 16 | **16p13.3** |
| ***PC*** | Pyruvate carboxylase | **11q13.2** |
| ***PCGF2*** | Polycomb group ring finger 2 | **17q12** |
| ***PDSS1*** | Decaprenyl diphosphate synthase subunit 1 | **10p12.1** |
| ***PEX1*** | Peroxisomal biogenesis factor 1 | **7q21.2** |

| | | |
|---|---|---|
| *PHF21A* | PHD finger protein 21A | **11p11.2** |
| *PHF6* | PHD finger protein 6 | **Xq26.2** |
| *PIGA* | Phosphatidylinositol glycan anchor biosynthesis class A | **Xp22.2** |
| *PIGM* | Phosphatidylinositol glycan anchor biosynthesis class M | **1q23.2** |
| *PIGN* | Phosphatidylinositol glycan anchor biosynthesis class N | **18q21.33** |
| *PIGT* | Phosphatidylinositol glycan anchor biosynthesis class T | **20q13.12** |
| *PIGV* | Phosphatidylinositol glycan anchor biosynthesis class V | **1p36.11** |
| *PIK3CA* | Phosphatidylinositol-4,5-bisphosphate 3-kinase catalytic subunit alpha | **3q26.32** |
| *PIK3R1* | Phosphoinositide-3-kinase regulatory subunit 1 | **5q13.1** |
| *PIK3R2* | Phosphoinositide-3-kinase regulatory subunit 2 | **19p13.11** |
| *PKDCC* | Protein kinase domain containing, cytoplasmic | **2p21** |
| *PLAG1* | PLAG1 zinc finger | **8q12.1** |
| *PLCB4* | Phospholipase C beta 4 | **20p12.3-p12.2** |
| *PLCH1* | Phospholipase C eta 1 | **3q25.31** |
| *PLG* | Plasminogen | **6q26** |
| *POC1A* | POC1 centriolar protein A | **3p21.2** |
| *POLE* | DNA polymerase epsilon, catalytic subunit | **12q24.33** |
| *POLR3A* | RNA polymerase III subunit A | **10q22.3** |
| *POMK* | Protein O-mannose kinase | **8p11.21** |
| *POMT1* | Protein O-mannosyltransferase 1 | **9q34.13** |
| *POP1* | POP1 homolog, ribonuclease P/MRP subunit | **8q22.2** |
| *PPP1CB* | Protein phosphatase 1 catalytic subunit beta | **2p23.2** |
| *PPP1R12A* | Protein phosphatase 1 regulatory subunit 12A | **12q21.2-q21.31** |
| *PPP2CA* | Protein phosphatase 2 catalytic subunit alpha | **5q31.1** |
| *PPP2R5D* | Protein phosphatase 2 regulatory subunit b'delta | **6p21.1** |
| *PTCH1* | Patched 1 | **9q22.32** |
| *PTCH2* | Patched 2 | **1p34.1** |
| *PTEN* | Phosphatase and tensin homolog | **10q23.31** |
| *PTPN11* | Protein tyrosine phosphatase non-receptor type 11 | **12q24.13** |
| *PUF60* | Poly(U) binding splicing factor 60 | **8q24.3** |
| *RAB39A* | RAB39A, member RAS oncogene family | **11q22.3** |
| *RAB39B* | RAB39B, member RAS oncogene family | **Xq28** |
| *RAC1* | Rac family small GTPase 1 | **7p22.1** |
| *RAF1* | Raf-1 proto-oncogene, serine/threonine kinase | **3p25.2** |
| *RALA* | RAS like proto-oncogene A | **7p14.1** |
| *RHEB* | Ras homolog, mTORC1 binding | **7q36.1** |
| *RIN2* | Ras and Rab interactor 2 | **20p11.23** |
| *RIT1* | Ras like without CAAX 1 | **1q22** |
| *RNF125* | Ring finger protein 125 | **18q12.1** |
| *RNF135* | Ring finger protein 135 | **17q11.2** |
| *ROR2* | Receptor tyrosine kinase like orphan receptor 2 | **9q22.31** |
| *RPS6KA3* | Ribosomal protein S6 kinase A3 | **Xp22.12** |
| *RRAS2* | RAS related 2 | **11p15.2** |
| *RXYLT1* | Ribitol xylosyltransferase 1 | **12q14.2** |
| *SATB2* | SATB homeobox 2 | **2q33.1** |
| *SEC23A* | SEC23 homolog A, COPII coat complex component | **14q21.1** |
| *SEC23B* | SEC23 homolog B, COPII coat complex component | **20p11.23** |
| *SEC24D* | SEC24 homolog D, COPII coat complex component | **4q26** |
| *SERPINH1* | serpin family H member 1 | **11q13.5** |
| *SETD2* | SET domain containing 2, histone lysine methyltransferase | **3p21.31** |
| *SGSH* | N-sulfoglucosamine sulfohydrolase | **17q25.3** |
| *SHANK3* | SH3 and multiple ankyrin repeat domains 3 | **22q13.33** |
| *SHOC2* | SHOC2 leucine rich repeat scaffold protein | **10q25.2** |
| *SIX3* | SIX homeobox 3 | **2p21** |
| *SLC25A1* | Solute carrier family 25 member 1 | **22q11.21** |
| *SLC2A1* | Solute carrier family 2 member 1 | **1p34.2** |
| *SLC2A10* | Solute carrier family 2 member 10 | **20q13.12** |
| *SLC44A1* | Solute carrier family 44 member 1 | **9q31.1-q31.2** |

| | | |
|---|---|---|
| ***SMAD4*** | SMAD family member 4 | **18q21.2** |
| ***SMO*** | Smoothened, frizzled class receptor | **7q32.1** |
| ***SNRPN*** | Small nuclear ribonucleoprotein polypeptide N | **15q11.2** |
| ***SNX10*** | Sorting nexin 10 | **7p15.2** |
| ***SNX14*** | Sorting nexin 14 | **6q14.3** |
| ***SON*** | SON DNA and RNA binding protein | **21q22.11** |
| ***SOS1*** | SOS Ras/Rac guanine nucleotide exchange factor 1 | **2p22.1** |
| ***SOST*** | Sclerostin | **17q21.31** |
| ***SOX9*** | SRY-box transcription factor 9 | **17q24.3** |
| ***SPINT2*** | Serine peptidase inhibitor, Kunitz type 2 | **19q13.2** |
| ***SPOP*** | Speckle type BTB/POZ protein | **17q21.33** |
| ***SPRED1*** | Sprouty related EVH1 domain containing 1 | **15q14** |
| ***SPRED2*** | Sprouty related EVH1 domain containing 2 | **2p14** |
| ***STRADA*** | STE20 related adaptor alpha | **17q23.3** |
| ***STT3A*** | STT3 oligosaccharyltransferase complex catalytic subunit A | **11q24.2** |
| ***SUFU*** | SUFU negative regulator of hedgehog signaling | **10q24.32** |
| ***SUZ12*** | SUZ12 polycomb repressive complex 2 subunit | **17q11.2** |
| ***SYN1*** | Synapsin I | **Xp11.3-p11.23** |
| ***SZT2*** | SZT2 subunit of KICSTOR complex | **1p34.2** |
| ***TAOK1*** | TAO kinase 1 | **17q11.2** |
| ***TBC1D2B*** | TBC1 domain family member 2B | **15q24.3-q25.1** |
| ***TBC1D7*** | TBC1 domain family member 7 | **6p24.1** |
| ***TBCK*** | TBC1 domain containing kinase | **4q24** |
| ***TBL1X*** | Transducin beta like 1 X-linked | **Xp22.31-p22.2** |
| ***TCF20*** | Transcription factor 20 | **22q13.2** |
| ***TCIRG1*** | T cell immune regulator 1, ATPase H+ transporting V0 subunit a3 | **11q13.2** |
| ***TET3*** | Tet methylcytosine dioxygenase 3 | **2p13.1** |
| ***THRA*** | Thyroid hormone receptor alpha | **17q21.1** |
| ***TMCO1*** | Transmembrane and coiled-coil domains 1 | **1q24.1** |
| ***TMEM53*** | Transmembrane protein 53 | **1p34.1** |
| ***TMEM165*** | Transmembrane protein 165 | **4q12** |
| ***TMEM216*** | Transmembrane protein 216 | **11q12.2** |
| ***TNFRSF11B*** | TNF receptor superfamily member 11b | **8q24.12** |
| ***TONSL*** | Tonsoku like, DNA repair protein | **8q24.3** |
| ***TPI1*** | Triosephosphate isomerase 1 | **12p13.31** |
| ***TRIM37*** | Tripartite motif containing 37 | **17q22** |
| ***TRIO*** | Trio Rho guanine nucleotide exchange factor | **5p15.2** |
| ***TRIP11*** | Thyroid hormone receptor interactor 11 | **14q32.12** |
| ***TRIP12*** | Thyroid hormone receptor interactor 12 | **2q36.3** |
| ***TSC1*** | TSC complex subunit 1 | **9q34.13** |
| ***TSC2*** | TSC complex subunit 2 | **16p13.3** |
| ***UPF3B*** | UPF3B regulator of nonsense mediated mRNA decay | **Xq24** |
| ***USP9X*** | ubiquitin specific peptidase 9 X-linked | **Xp11.4** |
| ***VPS35L*** | VPS35 endosomal protein sorting factor like | **16p12.3** |
| ***VPS4A*** | Vacuolar protein sorting 4 homolog A | **16q22.1** |
| ***WASHC5*** | WASH complex subunit 5 | **8q24.13** |
| ***WDR81*** | WD repeat domain 81 | **17p13.3** |
| ***WNT5A*** | Wnt family member 5A | **3p14.3** |
| ***XYLT1*** | Xylosyltransferase 1 | **16p12.3** |
| ***YME1L1*** | YME1 like 1 ATPase | **10p12.1** |
| ***ZBTB20*** | Zinc finger and BTB domain containing 20 | **3q13.31** |
| ***ZBTB42*** | Zinc finger and BTB domain containing 42 | **14q32.33** |
| ***ZBTB7A*** | Zinc finger and BTB domain containing 7A | **19p13.3** |
| ***ZDHHC9*** | Zinc finger DHHC-type palmitoyltransferase 9 | **Xq26.1** |
| ***ZIC2*** | Zic family member 2 | **13q32.3** |
| ***ZNF469*** | Zinc finger protein 469 | **16q24.2** |
| ***ZSWIM6*** | Zinc finger SWIM-type containing 6 | **5q12.1** |

**Figure S1**. Enriched biological processes of the gene set related to macrocephaly

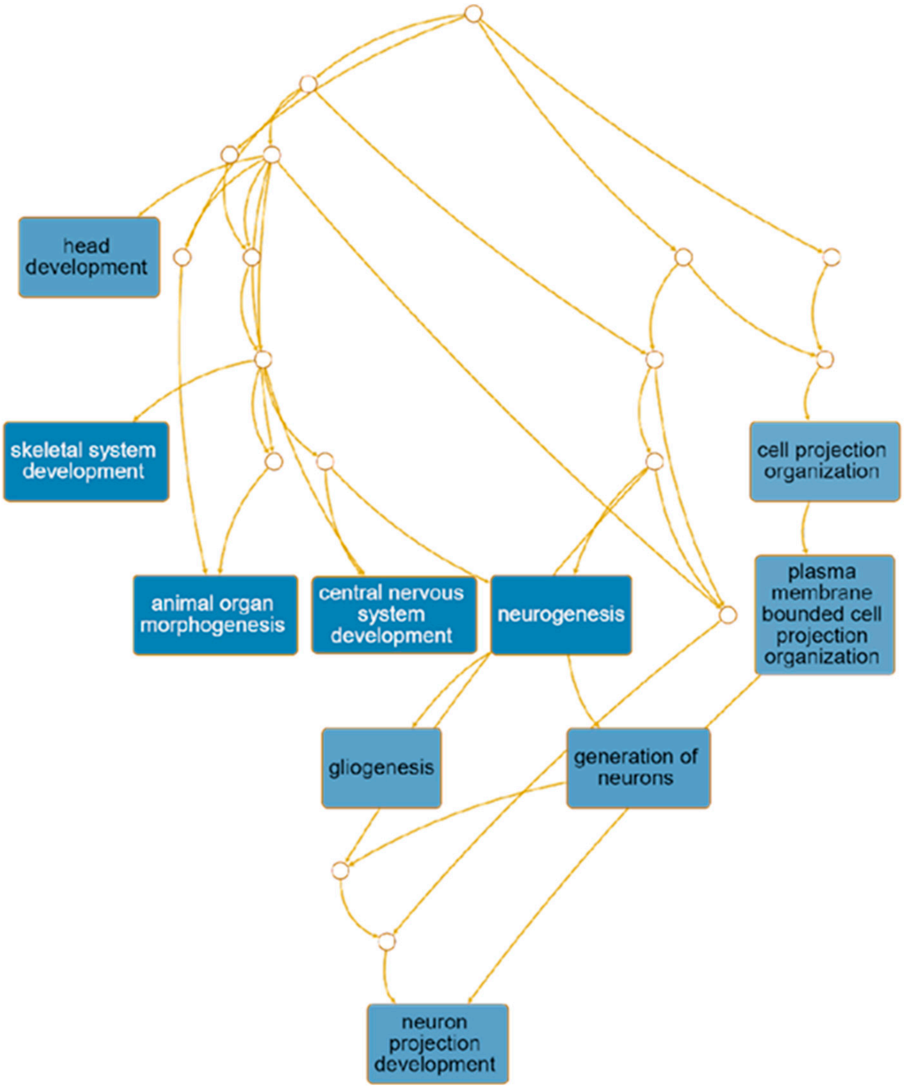

Supplement: Supplementary file 1 [file genes-13-02285-s001.zip › genes-2025175-supplementary.pdf]
