# Supplementary figures and images for: Rare CNVs and Known Genes Linked to Macrocephaly: Review of Genomic Loci and Promising Candidate Genes
Source: Genes (Basel). 2022 Dec 4;13(12):2285. doi: 10.3390/genes13122285 (PMC9778424; doi:10.3390/genes13122285)

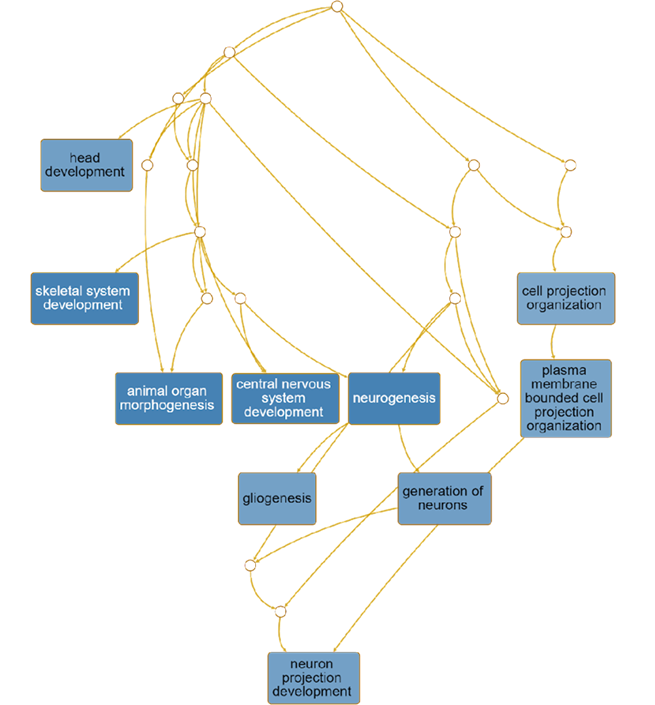

Supplement: Supplementary file 1 [file genes-13-02285-s001.zip › Supplementary_Figure_S1.tiff]
